# Whether interstitial space features were the main factors affecting sediment microbial community structures in Chaohu Lake

**DOI:** 10.3389/fmicb.2022.1024630

**Published:** 2022-12-14

**Authors:** Xiang Lu, Xiaotian Zhou, Christian von Sperber, Yaofei Xu, Zhipeng Wei, Siyan Li, Aidong Ruan

**Affiliations:** ^1^State Key Laboratory of Hydrology-Water Resources and Hydraulic Engineering, Hohai University, Nanjing, China; ^2^College of Hydrology and Water Resources, Hohai University, Nanjing, China; ^3^Department of Geography, McGill University, Montreal, QC, Canada

**Keywords:** sediment depth, total interstitial space, volumetric water content, gas space, particle features, microbial community structures

## Abstract

Sediments cover a majority of Earth’s surface and are essential for global biogeochemical cycles. The effects of sediment physiochemical features on microbial community structures have attracted attention in recent years. However, the question of whether the interstitial space has significant effects on microbial community structures in submerged sediments remains unclear. In this study, based on identified OTUs (operational taxonomic units), correlation analysis, RDA analysis, and Permanova analysis were applied into investigating the effects of interstitial space volume, interstitial gas space, volumetric water content, sediment particle features (average size and evenness), and sediment depth on microbial community structures in different sedimentation areas of Chaohu Lake (Anhui Province, China). Our results indicated that sediment depth was the closest one to the main environmental gradient. The destruction effects of gas space on sediment structures can physically affect the similarity of the whole microbial community in all layers in river dominated sedimentation area (where methane emits actively). However, including gas space, none of the five interstitial space parameters were significant with accounting for the microbial community structures in a sediment layer. Thus, except for the happening of active physical destruction on sediment structures (for example, methane ebullition), sediment interstitial space parameters were ineffective for affecting microbial community structures in all sedimentation areas.

## Introduction

Submerged sediments often exhibit strong microbial activities (greenhouse gas metabolism (Aben et al., 2017; Comer-Warner et al., 2019), nitrogenous compound metabolism (Reis et al., 2019; Liu and Yang, 2020; Tan et al., 2020), etc.) and are often biodegradation hotspots that balance global biogeochemical cycles (Mercier et al., 2014; Chen et al., 2016; Derrien et al., 2019). The relationships among sediment microbial community structure and environmental factors, such as pH, redox gradients (Ruuskanen et al., 2018), dissolved oxygen, total organic carbon content (Bryant et al., 2012), and sand features (Legg et al., 2012), have attracted much attention in recent years. Among these physicochemical factors, sediment interstitial space volume (porosity) can directly affect the absolute abundance of microbial communities, as it is the primary living space for sediment microorganisms (Hassard et al., 2017; Ahmerkamp et al., 2020). However, it is still not clear whether the features of the sediment interstitial space have effects on microbial community structures (Rebata-Landa and Santamarina, 2006; Scheidweiler et al., 2021).

The sediment interstitial space has two components, interstitial water and interstitial gas space. The structure of the interstitial space is initially sustained by sediment particles (Van Damme and Henri, 2018). The volume and structure of the interstitial space can be affected by particle features (sizes and evenness) (Mckinley et al., 2011; Dowey et al., 2017), gas emissions (Jain and Juanes, 2009; Lu et al., 2021), and microbial activities (cementation and mineralization) (Vorhies and Gaines, 2009; Gerbersdorf and Wieprecht, 2014). Thus, interstitial water content, interstitial gas space content, and particle features are all characteristics of the interstitial space of sediments. Recent studies have shown that sediment microbial community structures are strongly correlated with the sediment interstitial space volume (Li et al., 2020), particle sizes (Highton et al., 2016), and interstitial water content (Hollister et al., 2010; Zhang et al., 2021). These findings suggest that sediment interstitial space features may have effects on sediment microbial community structures. However, since the existing correlations are insufficient to confirm the bioeffects of the above sediment interstitial space features, further study is required.

In a stable sedimentary environment, sediment depth is typically the main direction of the environmental gradient. Environmental factors vary consistently along the depth direction, for example, TOC (Zan et al., 2011), dissolved oxygen (D'Hondt et al., 2015; Han et al., 2016), temperature (Biddle et al., 2011), pH (Nielsen et al., 2010), etc. Meanwhile, these environmental factors could significantly affect sediment microbial communities (Fierer and Jackson, 2006; Gilbert et al., 2012). As a result, it is common to find that sediment microbial communities usually vary consistently with sediment depth (Wang et al., 2016; Hiraoka et al., 2019). Since the sediment interstitial space features may also vary consistently along the sediment depth direction, it becomes harder to distinguish whether the correlations among interstitial space features and sediment microbial communities result from the bioeffects of the interstitial space features.

Therefore, the objectives of our study were (1) to determine the correlations of sediment interstitial space features (including total interstitial space volume, interstitial volumetric water content, interstitial gas space content, interstitial particle sizes, and evenness) with microbial community structure and, (2) to distinguish whether any discerned correlations have significantly affected microbial community structures. For this purpose, we analysed the abovementioned physicochemical parameters of sediments and calculated their correlations with the relative abundance of individual OTUs. Through comparison with sediment depth, the effects of these parameters on microbial community structures in sediments were determined. The results stressed the importance of analysing the interconnections among environmental factors with sediment depth in investigating their relationships with sediment microbial community sstructures.

## Materials and methods

### Environmental background and sediment sampling work

**Figure 1:**
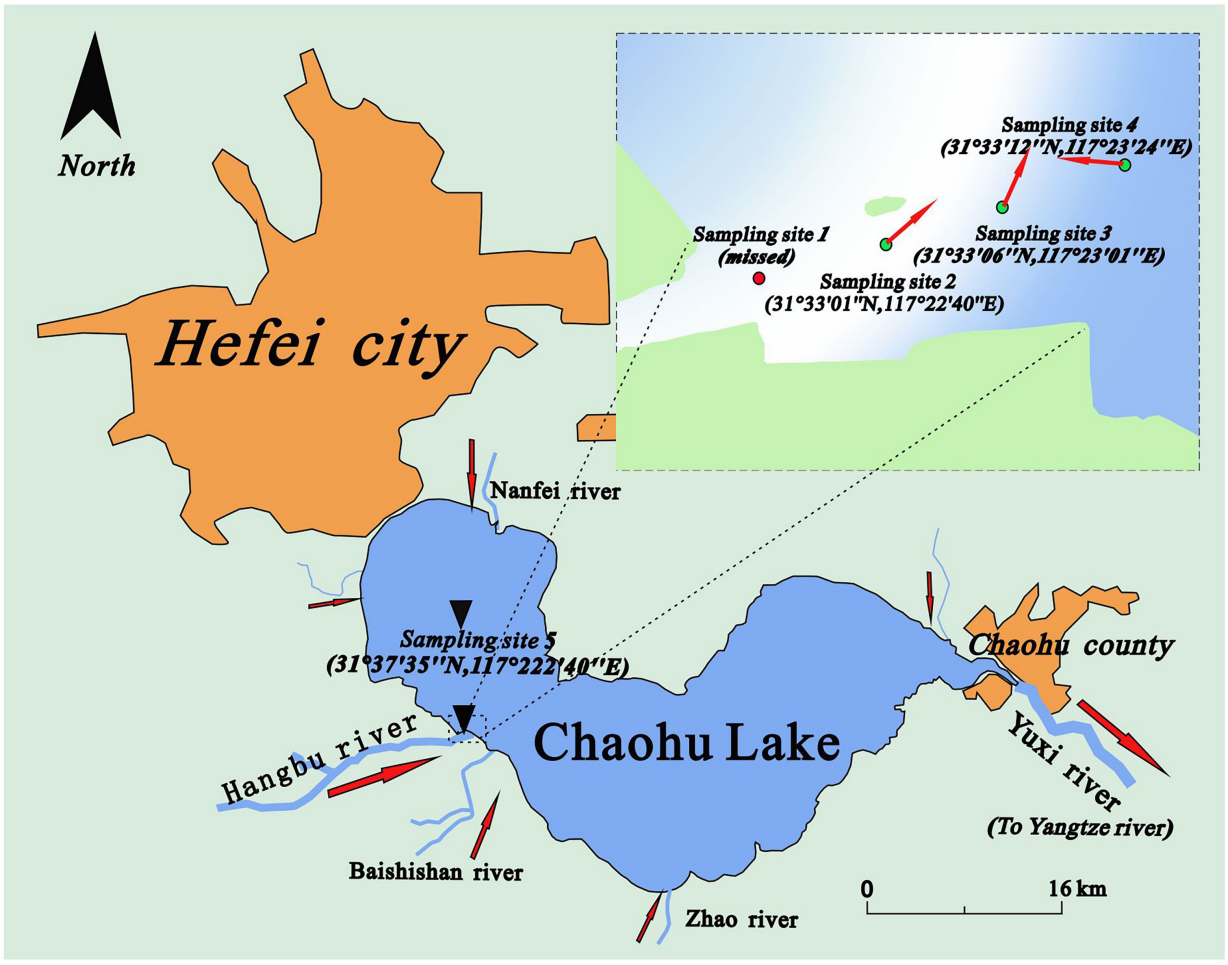
The locations of sampling sites (Sampling site 1 was upstream of site 2. The sediments there were nearly all composed of sand and could not be obtained by our sampling devices).

Chaohu Lake (560–825 km^2^, 16°C on average, 31°25′N-31°43′N, 117°17′E-117°50′E) is the fifth-largest shallow (2.8 m on average) freshwater lake in the Chang Jiang basin, China. It has 35 tributaries, of which the Hangbu River (length: 145 kilometres; area: 3064 km2) is the largest (3.06 billion m^2^ a year). Water quality assessments [in China Surface Water Quality Standards (GB3838-2002): Grade V (Yang et al., 2020)] show that Chaohu Lake is heavily eutrophic, especially in the western area (Shang and Shang, 2007). Compared with the western and northern surrounding areas, where more than 6 million people live with developed urban industries, the watershed of the Hangbu River consists of agricultural fields. The water quality of the Hangbu River (Grade II) is also much less impacted than the western lake (see Figure 1).

In this study, an *in situ* sampler equipped with a heavy hamper was used to obtain sediment samples at four sampling sites along a transect from the Hangbu estuary to the western part of central Chaohu Lake on June 2, 2019. To improve the representativeness of this study, the four sampling sites were set in three kinds of sedimentation areas (River dominated, transition area, and lake dominated). Principles of dividing different sedimentation areas were their changes of hydraulic conditions (velocity, flow direction) and particle properties. The detailed description and discussion of this part can be seen in our previous study (Lu et al., 2021). As a result, the intervals of sampling sites 2, 3, and 4 were all approximately 600 metres. Sampling site 5 was in the centre of the western part of Chaohu Lake. The distance between site 4 and site 5 was approximately 8.5 kilometres. The water depths were 2.25 m, 2.55 m, 1.30 m and 3.00 m at sampling sites 2 ~ 5. Four sampling tubes with sediment were obtained by an *in situ* sampler equipped with a heavy hamper (the structure of the sampler can be seen in the Supplementary files of our previous studies (Lu et al., 2021)). During the short transportation time (less than 2 h), ice bags and an insulated cabinet were used to keep the sediments at a stable status. The depths of the four sediment columns varied from 45 cm to 60 cm. After they were transported into the laboratory, the sampling tube was divided vertically, and the sediment columns were immediately separated into 5 cm sections (Reasons for setting 5 cm as a layer were that it can smoothly showed the changes of microbial community structures and met the limitations of the measuring device (TR-6D)). During the dissection process, physicochemical parameters such as volumetric water content, and other parameters (later description.) were measured at each sediment layer. The sediment sample at each 5 cm layer was placed into a sterile plastic bag and squeezed evenly before it was divided into five small plastic bags for testing different parameters. The divided sediment samples that were used for sequencing were stored in a refrigerator (−80°C). In addition, for each sampling location, 36 sediment samples obtained from the top 9 layers (0–45 cm) were selected for further analysis. More details of the sampling protocol can be found in a previous study (Lu et al., 2021).

### Data collection of sediment interstitial space features

Six environmental factors, including sediment depth (cm), volumetric water content (%), total interstitial space percent (%), gas space percent (%), average particle size (μm), and particle evenness, were measured at each of the 5 cm increments.

The volumetric water content $\left(Moi_{\left(v\right)}\right)$ was measured using a soil water content meter (TR-6D, Shunkeda, Beijing, China). Its physical measurement was determined as follows:


(1) $$Moi_{\left(v\right)}=\frac{V_{\left(w\right)}}{V_{\left(T\right)}}=\frac{V_{\left(w\right)}}{V_{\left(s\right)}+V_{\left(w\right)}+V_{\left(a\right)}}$$

$V_{\left(w\right)}$ is the volume of the pore water. $V_{\left(s\right)}$ is the volume of the solid particles. $V_{\left(a\right)}$ is the volume of the gas space. $V_{\left(T\right)}$ represents the total volume of the sediment layer sample.

The percentage of layered gas space volume ( $VP_{\left(a\right)}$ ) was calculated as follows (Lu et al., 2021):


(2) $$VP_{\left(a\right)}=1-\frac{Moi_{\left(v\right)}•\rho_{\left(w\right)}}{Moi_{\left(m\right)}•\rho_{\left(w\&s\right)}}$$

$\;VP_{\left(a\right)}$ is the gas space volume percent. $\rho_{\left(w\right)}$ is the pore water density, which was measured gravimetrically. $Moi_{\left(m\right)}$ is the sediment mass water content, which was measured by the drying method described in (Lu et al., 2021). $\rho_{\left(w\&s\right)}$ represents the density of mixed sediment and was measured by the submerged method (Lu et al., 2021). The data of each layered sediment sample consisted of the average value of five replicates.

The total interstitial space volume percentage was the sum of the gas space volume percentage and volumetric water content. The equation is as follows.


(3) $$TIS=Moi_{\left(v\right)}+VP_{\left(a\right)}$$

The average particle sizes were measured by a laser particle sizer (LS13320, Beckman Coulter, Brea, CA, United States). Particle evenness was the coefficient of variation (Cv). Similarly, each layered sample had five replicates.

### DNA extraction and high-throughput sequencing

The homogenized sediment samples that were stored in the refrigerator (−80°C) were freeze-dried. Then, three subsamples of 250 mg freeze-dried sediment from each layer were weighed for DNA extraction, and a PowerSoil DNA Isolation Kit (QIAGEN, Carlsbad, United States; previously from MoBio Laboratories Inc.) was used. The absorbance ratio of DNA subsamples at OD260/280 and OD260/230 were controlled under the range of 1.7–1.9 and 2.0–2.5, respectively. DNA subsamples that could not satisfy the above conditions were extracted again. After extraction, three qualified DNA subsamples of each layer were mixed and used for PCR amplification. Here, a pair of universal primers, 515F (5’-GTGYCAGCMGCCGCGGTAA-3′) and 926R (5’-CCGYCAATTYMTTTRAGTTT-3′), were used to amplify the V4-V5 variable region of 16Sr RNA to detect bacteria and archaea (Alma et al., 2015). In addition, extraction kit elution buffer was used as a negative control. The PCR cycling procedure was set as follows: 5 min for initial denaturation at 95°C, followed by 25 cycles of 95°C for 30 s, 50°C for 45 s, 68°C for 90 s, and a final extension at 68°C for 10 min. Then, the amplified DNA products were purified by using the OMEGA DNA purification kit (Omega Bio-Tek Inc., Doraville, GA, USA) and further purified by electrophoresis in agarose gels before using the Monarch® DNA gel Extraction Kit (New England Biolabs, USA) for gel extraction. Finally, the PCR products were sent for high-throughput sequencing on an Illumina HiSeq2500 platform (2 × 250 paired ends, Illumina, San Diego, USA) at the Biomarker Technologies Corporation, Beijing, China. The number of layered sediment samples was 36 in total.

During the process of controlling the quality of the original sequencing data (2,880,313 reads in total), raw sequence data (2,772,268 tags) were merged with FLASh v1.2.7 (Magoc and Salzberg, 2011), which removed sequences whose length was less than 250 bp. Then, Trimmomatic v0.33 (Bolger et al., 2014) was used to filter the low-quality sequence data (detection: 50 bp; quality: less than 20) and obtain clean tags (2,727,994). UCHIME v4.2 (Edgar et al., 2011) was used to overlap the PE reads, filter and obtain high-quality sequences (2,680,295) (threshold of chimaeras: 80% similarity). The OTUs (operational taxonomic units) were clustered at 97% similarity by using Usearch (Edgar, 2013), and those whose relative abundance was less than 0.005% were filtered (residual tags: 1,813,882). Based on the Silva database (release 128, http://www.arb-silva.de), the residual OTUs (2,219 in total) were annotated. Further, to avoid the effects of non-microbiota on subsequent analysis, chloroplast and mitochondria were removed by QIIME (Zhang et al., 2019). At last, the residual OTUs (2,210 in total) was standardized and resampled by using package vegan (2.6.2, function: rrarefy) in R Language. Besides, detailed information on sequence quality control and a summary of annotation (taxonomy) at each layer of different sampling sites can be seen in Supplementary Table S1. The detailed OTU table can be seen in an Excel file (Supplementary files).

Raw joined sequence data were uploaded to the NCBI SRA database (Number: PRJNA482178; Sample data: SAMN15746538 - SAMN15746549 (Sampling site 5) and SAMN15746556 - SAMN15746585 (Sampling site 2–4); Link: https://www.ncbi.nlm.nih.gov/bioproject/?term=prjna482178).

### Statistical analysis

**Figure 2:**
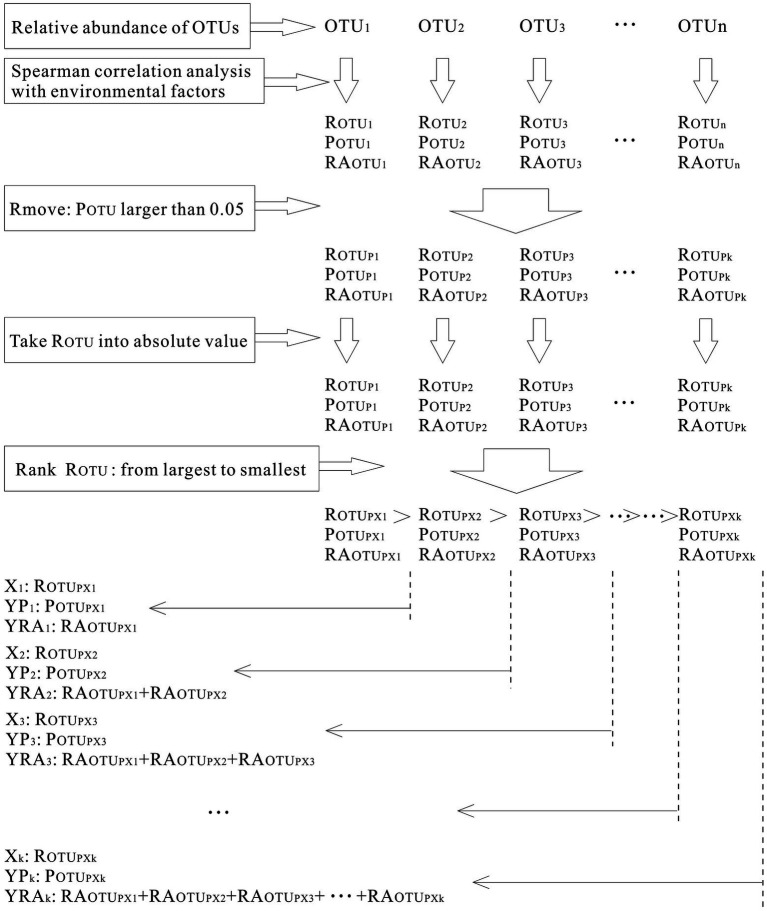
Processes of calculating microbial community’s correlation coefficients with environmental factors.

**Figure 3:**
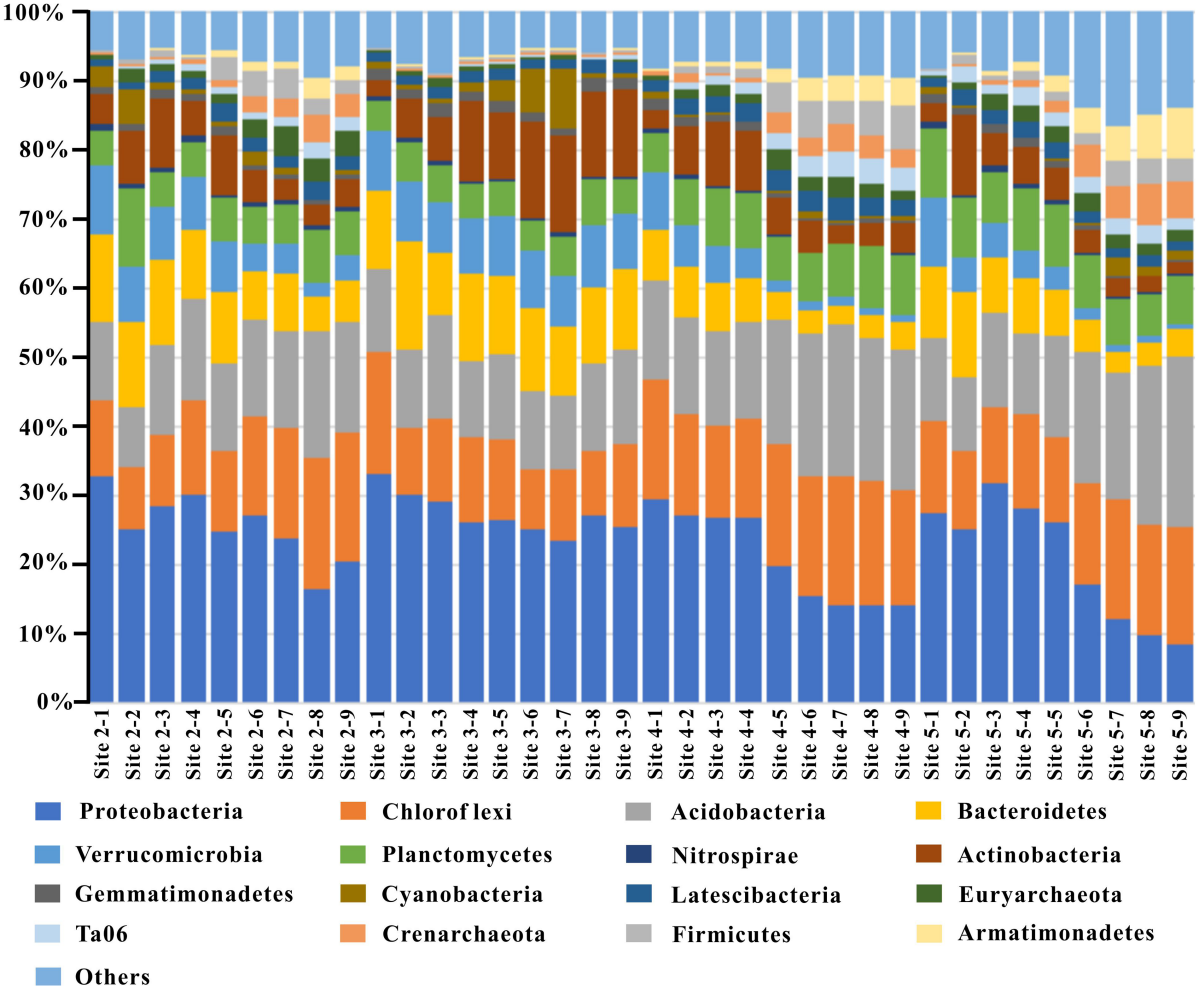
The top ten phyla at each sediment layer.

**Figure 4:**
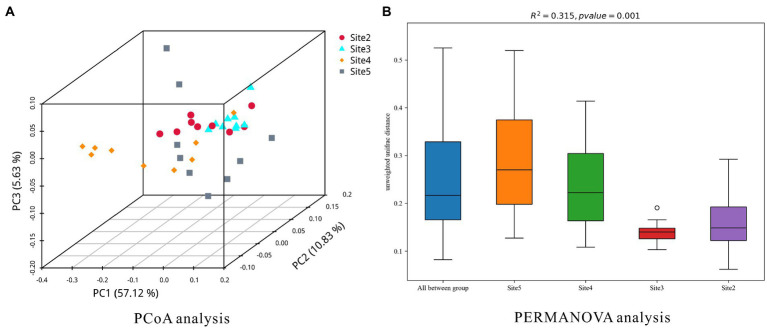
The dissimilarities of the microbial community at each sampling site.

At each layer of the four sampling sites, the relative abundance of each OTU was calculated (RA_OTU_). Then, Spearman correlation analysis was applied among the above six environmental factors (sediment depth, volumetric water content, total interstitial space, gas space, particle size, and particle evenness) and the relative abundance of OTUs at each sampling site. According to the results, those OTUs whose significance (P_OTU_) was larger than 0.05 were removed. Since this analysis aimed to calculate which OTUs exhibited correlations with environmental factors, regardless of positive correlations or negative correlations, they were counted together. Thus, correlation coefficients (R_OTUs_) were converted into absolute values and ranked from largest to smallest. Furthermore, for each environmental factor, to see how their correlated OTUs varied with correlation coefficients, the scatter figure of correlation coefficients versus significance (X_1_ vs. YP) and relative abundance of correlated OTUs (X_1_ vs. YRA) were calculated and plotted (Figure 2). The process is shown in Figure 3. Additionally, to examine the significance of the *p*-value in Figure 4, FDR (False discovery rate) was been set as lower than 5% and it was been calculated in the way of Benjamini – Hochberg. PCoA analysis was conducted in R language (Package: vegan 2.6.2; Function: cmdscale; Distance settings: unweighted unifrac). PERMANOVA analysis was performed in R language (Package: vegan 2.6.2; Function: Adonis; Distance settings: unweighted unifrac; Permutations: 999) and plotted in Python. RDA analysis was conducted and plotted in R language (Package: Vegan 2.6.2; settings: rda(scale = FALSE), using permute to calculate significance).

## Results

### Distributions of the sediment interstitial space features

The measurement data are briefly summarized in Table 1 (more details can be seen in Supplementary Table S1). From the Hangbu estuary to the western Chaohu Lake centre, the values of the total interstitial space percentage ranged from 56.1 to 88.2%. Except for sampling site 3, the percent values decrease with depth. In addition, compared to the estuary area, interstitial space volumes were much larger in the central lake. Volumetric water content values ranged from 49.3 to 88.2% and were similarly distributed with interstitial space percent. Gas space only continuously existed in the Hangbu estuary and ranged from 0 to 17.93%. The distributions bore no relationships with sediment depth. The average values of particle sizes at each sampling site ranged from 7.32 μm to 338.6 μm and decreased sharply from the Hangbu estuary to the lake center. The vertical fluctuations in the particle size distributions at the four sampling sites were much larger the closer the sample site was to the upstream reaches of the Hangbu estuary. In addition, the particle evenness of each sampling site ranged from 0.76 to 1.64, and its values in the central lake were much larger than those in the estuary area.

**Table 1:** The detailed data of interstitial space features.

Sediment depth (cm)	Sampling site 2					Sampling site 3					Sampling site 4					Sampling site 5				
	TIS (%)	VWC (%)	*G*-space (%)	*P*-size (μm)	*P*-evenness	TIS (%)	VWC (%)	*G*-space (%)	*P*-size (μm)	*P*-evenness	TIS (%)	VWC (%)	*G*-space (%)	*P*-size (μm)	*P*-evenness	TIS (%)	VWC (%)	*G*-space (%)	*P*-size (μm)	*P*-evenness
2.5	72.4	65.7	6.67	43.99	1.00	67.9	67.9	0	37.68	1.12	67.4	67.4	0	30.77	1.10	88.2	88.2	/	9.52	1.39
7.5	75.5	59.5	16.03	28.87	0.87	76.8	58.9	17.93	67.90	1.38	69.2	64.5	4.73	23.95	1.09	88.2	88.2	/	8.56	1.47
12.5	72.0	60.9	11.09	23.99	0.92	63.6	58.1	5.52	69.29	1.00	69.9	63.3	6.62	27.60	1.22	84.9	84.9	/	11.13	1.64
17.5	62.1	55.3	6.81	71.58	0.94	61.8	55.3	6.53	64.32	1.20	69.6	59.5	10.05	29.31	1.11	73.3	73.3	/	13.92	1.55
22.5	66.5	53.3	13.19	42.40	1.16	65.6	61.6	4.01	39.58	1.14	61.3	54.4	6.92	25.25	0.95	64.4	62.0	2.4	14.32	1.61
27.5	- -	- -	- -	338.60	0.76	70.7	62.9	7.79	40.27	1.11	62.4	55.3	7.09	21.30	0.99	61.2	61.2	/	14.76	1.61
32.5	- -	- -	- -	162.48	1.02	67.6	60.2	7.43	34.98	1.06	59.3	54.2	5.10	21.58	1.00	64.4	60.0	4.4	13.31	1.57
37.5	60.6	51.8	8.82	62.13	1.37	70.5	61.4	9.07	32.50	1.18	59.0	56.1	2.91	16.62	0.97	65.7	60.7	5.0	7.32	0.91
42.5	57.6	49.3	8.28	66.55	1.30	65.2	62.6	2.57	30.82	1.10	56.1	53.6	2.49	15.13	0.94	61.8	61.8	/	10.23	1.30
Water depth (cm)	225					255					130					300				
Temperature (°C)	24.6 ~ 25.2					24.7 ~ 25.2					27.1 ~ 28.0					24.1 ~ 24.4				

TIS, Total interstitial space percent; VWC, Volumetric water content; *G*-space, Gas space percent, *P*-size, Particle average size; *P*-evenness, Particle evenness. The notation of “- -” refers to the missing data (During the measurement, the sediment layer was too dry and broken by the instrument probes). In addition, no continuous gas space data was observed in the lake later (Sampling site 5).

### Components and dissimilarities of the sediment microbial community

**Figure 5:**
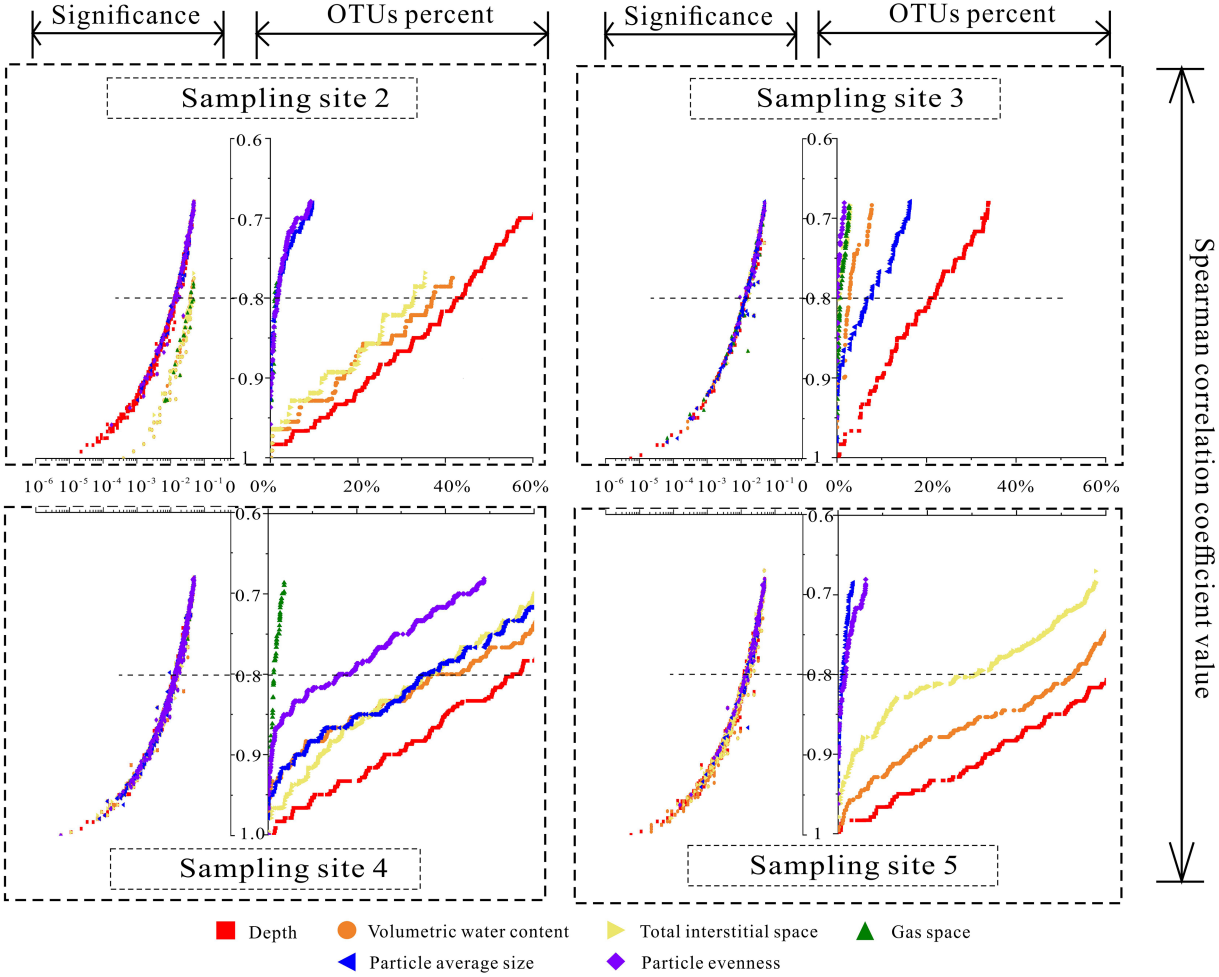
The variations of the correlations among interstitial space features and relative abundance of correlated OTUs.

According to the sequence data of 16S rRNA, the relative abundances of the top ten phyla at each layer of the sampling site were plotted (Figure 5). Proteobacteria (8.73% ~ 33.28%), Acidobacteria (8.60% ~ 24.71%), Chloroflexi (8.72% ~ 18.89%), Bacteroidetes (2.82% ~ 15.43%), Planctomycetes (4.43% ~11.37%), Verrucomicrobia (0.71% ~ 10.04%), and Actinobacteria (1.87% ~ 14.15%) were the abundant phyla that were shared among all sampling sites. In contrast, Nitrospirae (0.98% ~ 6.18%), Gemmatimonadetes (0.12% ~ 1.86%), Cyanobacteria (0.10% ~ 8.59%), Latescibacteria (1.18% ~ 3.42%), Euryarchaeota (0.24% ~ 4.12%), TA06 (0.09% ~ 3.72%), Crenarchaeota (0.10% ~ 5.89%), Firmicutes (0.14% ~ 6.31%), and Armatimonadetes (0.06% ~ 7.36%) were only abundant in some sediment layers.

Besides, except for the distributions at sampling site 3, the relative abundance of the above phyla varied with depth at the other three sites. For example, at sampling sites 2, 4 and 5, the relative abundances of Proteobacteria, Bacteroidetes, Actinobacteria, Verrucomicrobia, Gemmatimonadetes and other phyla varied negatively with increasing sediment depth. Meanwhile, the relative abundances of Chloroflexi, Acidobacteria, TA06, Armatimonadetes, and other phyla were positive with increasing sediment depth. However, at sampling site 2, except for Proteobacteria (negative), Actinobacteria (positive) and Cyanobacteria, the relative abundances of the other phyla were insensitive to increasing sediment depth. Overall, from site 5 to site 4, site 2, and site 3, the relative abundance of multiple phyla invariably increased with increasing depth; for instance, Chloroflexi, Acidobacteria, Bacteroidetes, Verrucomicrobia, TA06, Armatimonadetes, etc.

**Figure 6:**
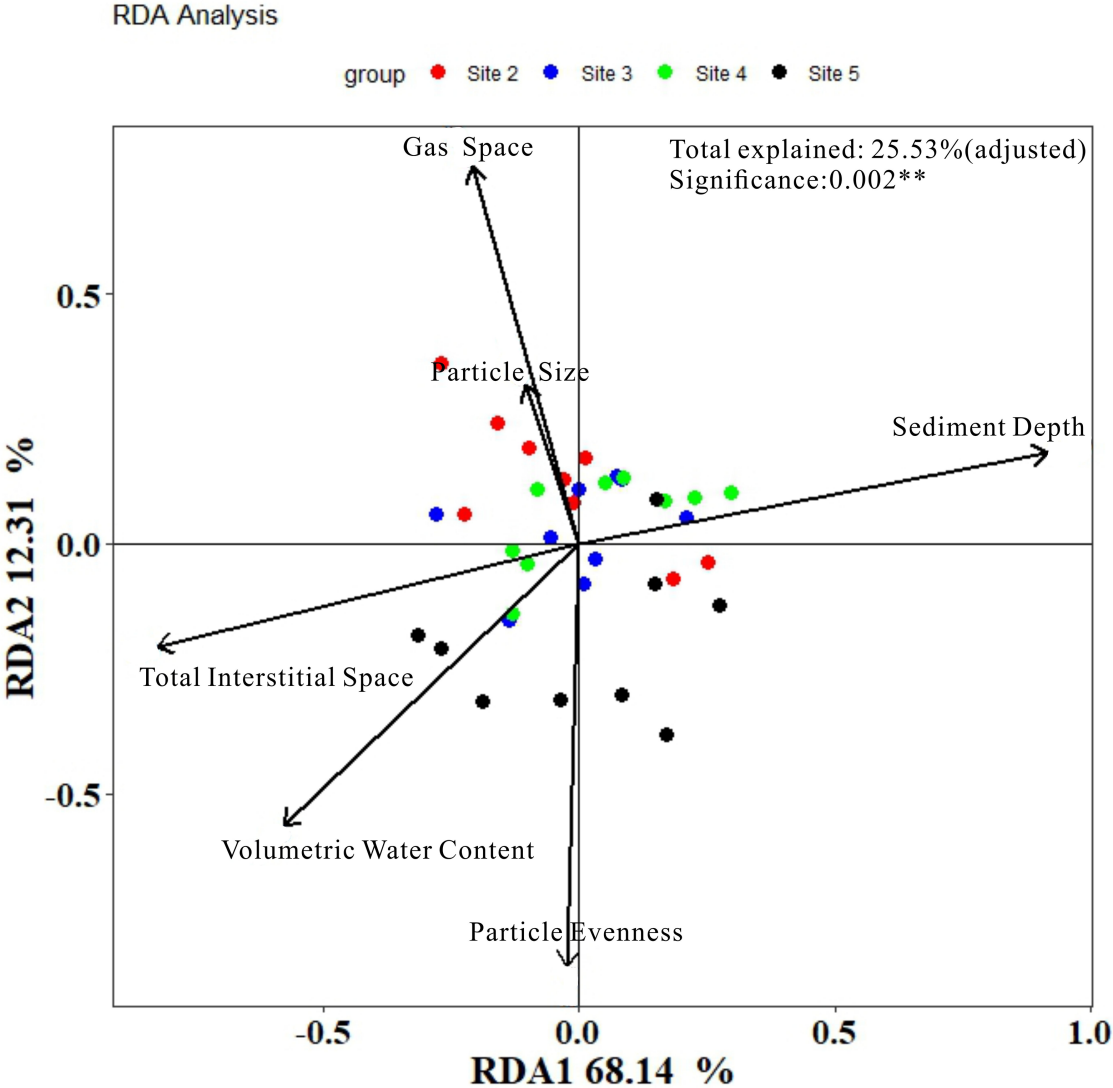
Results of RDA analysis.

In addition, PCoA and PERMANOVA analysis were applied to investigate the dissimilarities of the microbial community at each sampling site. The results were plotted as Figure 6. The results of them indicated that the similarity of microbial community at sampling site 3 was the highest one. The similarity of microbial community at sampling site 5 was the lowest one. In the results of PCoA analysis, space distances of different sediment layers were showed (Site 5 > Site 4 > Site 2 > Site 3). In the result of PERMANOVA analysis, the similarities of microbial community structures in different sediment layers at the four sites (chamber size, Site 3 > Site 2 > Site 4 > Site 5) were in accordance with the methane bubble (gas space) emission stages (emission stage: Site 3 > Site 2 > Site 4 > Site 5).

### Correlations among sediment interstitial space features and the microbial community

Based on the results of Spearman correlation analysis among individual OTUs and six environmental parameters, the variations in the relative abundance of the correlated OTUs with Spearman correlation coefficients were plotted at four sampling sites (Figure 2). According to the effective relative abundance of the correlated OTUs, when Spearman correlation coefficient values were larger than 0.8, even at sampling site 3, the distributions of more than 20.81% of the total OTU community correlated with sediment depth. At sampling site 5, the relative abundance was more than 62.25%. Sediment depth showed the strongest correlation (more correlated OTUs) with the communities at all sampling sites. In contrast, the correlation of the gas space volume was the weakest. Meanwhile, for the other four environmental factors, the relative abundance of their correlated OTUs varied with different sampling sites. Especially at sampling site 4, except for the gas space, more than half of the total community was correlated with other environmental factors.

Additionally, when the Spearman correlation coefficient was larger than 0.8, the components of the correlated OTUs of each environmental factor were plotted (Figure 4). From that figure, for sediment depth, the main correlated OTUs came from Proteobacteria (23.28% on average), Acidobacteria (17.79%), Chloroflexi (14.02%), Bacteroidetes (9.55%), Actinobacteria (6.43%), and Planctomycetes (6.55%). For volumetric water content, the main correlated phyla included Proteobacteria (18.18%), Acidobacteria (15.44%), Chloroflexi (14.17%), Planctomycetes (10.24%), Bacteroidetes (5.31%), and Nitrospirae (14.63%, only abundant in site 3). The total interstitial space included Proteobacteria (19.98%), Chloroflexi (16.67%), Acidobacteria (16.64%), Planctomycetes (10.60%), and Bacteroidetes (8.50%). For gas space, the main correlated OTUs were Proteobacteria (34.91%), Chloroflexi (17.68%), Acidobacteria (7.21%), Bacteroidetes (11.61%, not exist in site 2), Actinobacteria (8.18%, not exist in site 2), Rokubacteria (23.36%, only exist in site 4), and Nitrospirae (17.20%, only exist in site 2). For particle size, the main correlated OTUs were Proteobacteria (28.29%), Acidobacteria (18.35%), Chloroflexi (8.02%), Planctomycetes (7.05%), and Nitrospirae (8.73%). For particle evenness, the main correlated OTUs were Proteobacteria (37.55%), Planctomycetes (12.36%), Acidobacteria (9.56%), and Chloroflexi (12.77%, not exist in site 2). In addition, there were special correlated phyla that only existed in selected environmental niches. Specifically, Cyanobacteria only existed in the correlated phyla of the total interstitial space. Omnitrophicaeota was only existed in the correlated phyla of particle size and evenness.

**Figure 7:**
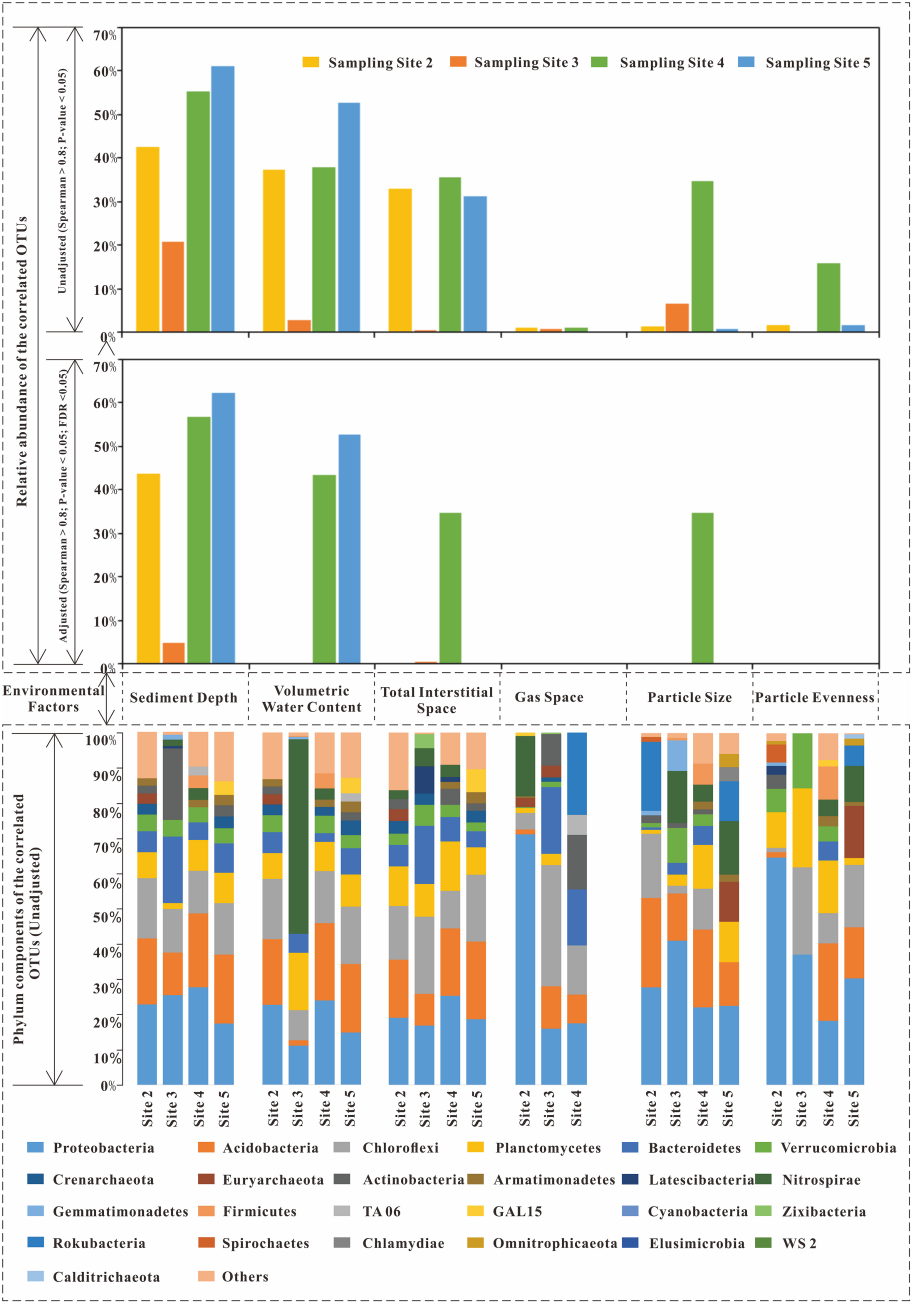
The correlated OTUs of six environmental factors (Spearman correlation coefficients larger than 0.8).

To compare the results of our method with the results of classical method, RDA analysis was conducted and the results were plotted in Figure 7. As it showed, the model explained value was only 38.30% in total (after adjusted was 25.53%). Axis of RDA1 and RDA2 were, respectively, account for 68.14 and 12.31% of the total model explained value. The results of the detailed information about each environmental factors can be seen in Table 2.

**Table 2:** Results of RDA analysis (detailed information).

Factors	RDA1	RDA2	*R*^2^ (explained)	Adjusted *R*^2^	*p*
Depth	0.91887	0.39457	0.5667	0.37779231	0.001
TIS	−0.9047	−0.42604	0.4764	0.31759353	0.001
VWC	−0.71905	−0.69495	0.4786	0.31906017	0.001
*G*-space	−0.02073	0.99979	0.3048	0.20319586	0.003
*P*-size	−0.07924	0.99686	0.0544	0.03626593	0.392
*P*-evenness	−0.28072	−0.95979	0.4178	0.27852766	0.001

From Figure 7 and Table 2, the accountable level of the six environmental factors can be ranked as: sediment depth (significant) > Volumetric Water Content (significant) > Total Interstitial Space (significant) > Particle evenness (significant) > Gas space (significant) > Particle average size (insignificant). Thus, when we regardless of the different results in particle features, to some extent, the results of RDA analysis were similar to our correlation analysis, which based on individual OTUs. However, no matter RDA analysis or our correlation analysis, they were all statistics results. Given the possible effects of collinearity, more discussion shall be conducted to investigate whether these environmental factors have effects on the microbial community in a layer (or in a site column).

## Discussion

### Analysis of sediment depth and gas space with their correlated OTUs

In our study, sediment depth had the highest relative abundance of the correlated OTUs compared with other environmental factors at all sampling sites (except for sampling site 3, Figures 4, 7). In other words, among these six environmental factors, sediment depth was the closest to the main direction of the sediment environmental gradient. In addition, in Chaohu Lake sediment, other studies also showed that the vertical distributions of many environmental factors were consistent with sediment depth, for example, TP (total phosphorus content), TOC (total organic carbon content) (Zan et al., 2011), TN, Pb (Chen et al., 2013), etc. Since sediment depth was just a spatial direction with no direct bioeffects on microbes, it can be used as a representative of the main environmental gradient to analyze its relationships with the other five environmental factors and microbial community structures.

However, the correlated OTUs of sediment depth varied apparently with different sampling sites (21% ~ 62%). There probably some unknown factors which impeded sediment depth to become the main environmental gradient at all sites. As the results showed in Figure 4, from abundant to less, the correlated OTUs of sediment depth decreased sharply from site 5 to site 4, site 2 and site 3. The decreasing order was in accordance with the increasing order of the methane bubble emission stage (Site 2: emission stage II; Site 3: emission stage II to III; Site 4: emission stage I; Site 5: not happened) we have studied before (Lu et al., 2021). Given that gas space formation (methane emissions (Chen and Slater, 2016; Lu et al., 2021)) can destruct sediment structures and promote excess pore water exchange in different layers (Lu et al., 2021), the microbes in pore water probably could be carried away and moved to other layers. As a result, with the destruction effects of gas space become stronger, the community similarities in different layers would subsequently become larger.

**Figure 8:**
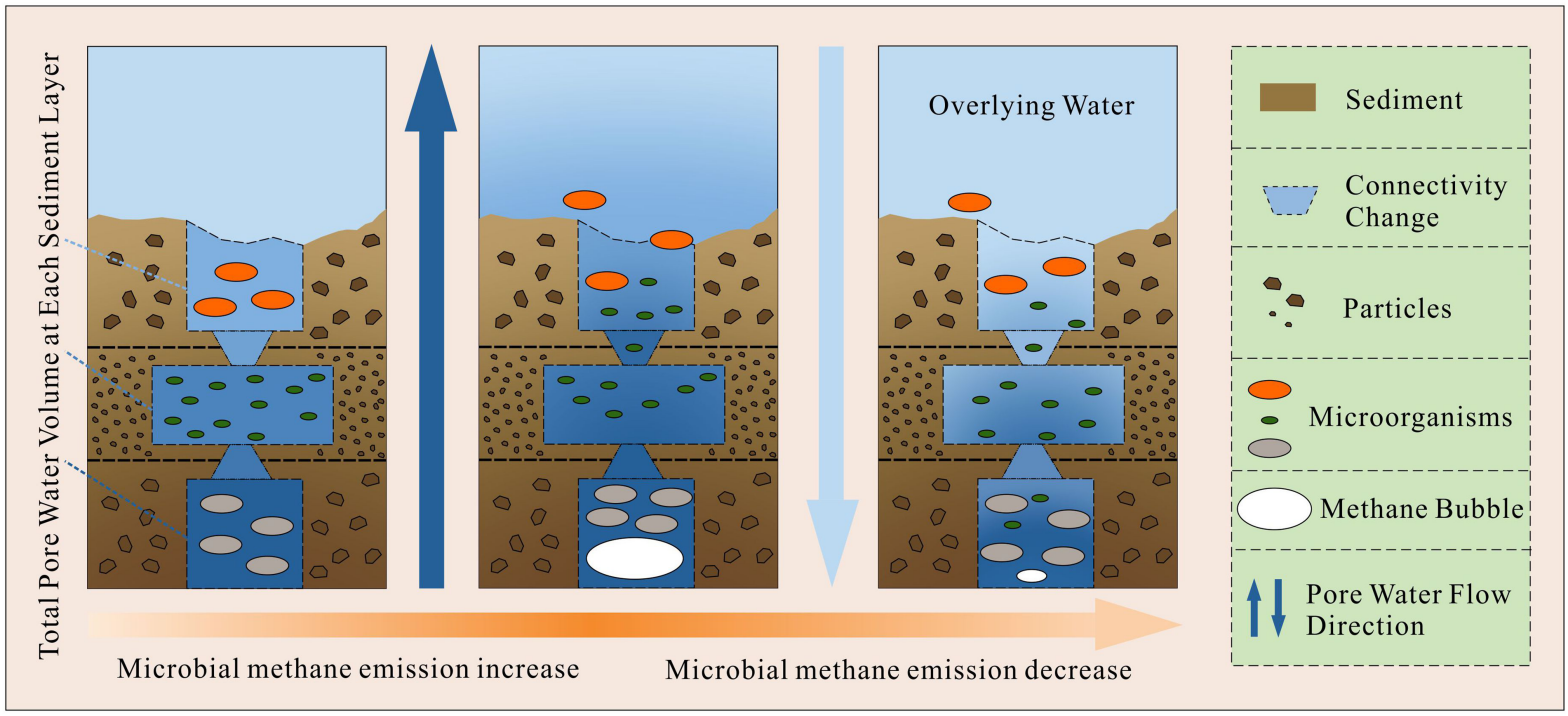
The theoretical processes of how methane bubble variations affect sediment microbial communities along depth direction.

To confirm the above assumption, five pieces of evidence can be used to supported it. First, our previous study (Lu et al., 2021) concluded that the gas space formed by excess methane emissions can change the sediment interstitial space and pore water exchange (up to 17%), so it could physically move the microbes in pore space. Second, from Figure 5, with the increase in methane emission stages, the relative abundance of main phyla became more even along the sediment depth direction (for example, Proteobacteria, Chloroflexi, Acidobacteria, Bacteroidetes, Verrucomicrobia, Planctomycetes, etc.). Third, the distributions of the phyla with larger cell sizes were limited, but the smaller cell sizes of the OTU phyla became more even as the methane emission stage increased. The above phenomenon was in accordance with the description in Figure 8. For example, Cyanobacteria live in the underlying water, and their cell sizes are usually larger than those of other bacteria (smallest cyanobacteria: Picocyanobacteria (Jasser and Callieri, 2017), 0.70 ± 0.46 μm^3^ on average (cell size of different classes multiply the frequency of different classes), (Albertano et al., 1997)). The distributions were limited in the sediment layers where the total interstitial space was large (Figure 5; Table 1). Moreover, the cell size of Proteobacteria is usually smaller (0.03 μm^3^ on average, Delaware estuary, (Cottrell and Kirchman, 2004)) than that of other bacteria (Krieg et al., 2010). The relative abundance became more even (from site 2 to 5, standard deviation: 4.9, 3.0, 6.6, and 8.8%) with increasing emission stage, and none of the relative abundances correlated with the total interstitial space (site 3, Figure 4). Thus, if gas space variations help mix the microbial communities at different layers, the transport of those larger cell size bacteria (Cyanobacteria) and smaller size of bacteria will satisfy the above descriptions. Fourth, Figure 4 shows that the phyla of correlated OTUs varied with different sampling sites. They are not methanogens or methanotrophs and have motilities or filiform structures to resist pore water exchange, for example, Bacteroidetes (Prolixibacteraceae (Watanabe et al., 2020)), Actinobacteria (Fernández-Gómez et al., 2013; Anandan et al., 2016), and Chloroflexi (Anaerolineae (Yamada et al., 2006)). Fifth, the decrease of the similarities (the kinds and contributions of the correlated phyla between sediment depth and volumetric water content) was in accordance with the changes of methane emission stage. (More details have been discussed in Section 4.3.)

Overall, gas space may not be the effective factor to account for the community structure in a layer, but it can affect the similarities of microbial community structure among different layers of a sampling site.

### Analysis of particle features (size and evenness) with their correlated OTUs

RDA analysis and correlation analysis all showed that particle features were not the main effective factor to sediment microbial community structures at all sampling sites. However, to investigate why the correlated OTUs of particle features vary markedly with different sampling sites. Comparisons between particle features and the main environmental gradient (depth) were discussed. (Sediment depth was not the main environmental gradient at site 3, so comparison at site 3 was been removed.)

Compared with sediment depth, particle size had similar correlated phyla with similar abundance components (Figure 4, sediment depth: Proteobacteria (23.28%), Acidobacteria (17.79%), Chloroflexi (14.02%), and Planctomycetes (6.55%); particle size: Proteobacteria (28.29%), Acidobacteria (18.35%), Chloroflexi (8.02%), Planctomycetes (7.05%)). Moreover, from site 2 to site 5 (site 3 was removed), the Spearman correlation coefficients between sediment depth and particle size were 0.33, −0.89, and 0.07, respectively. The coefficient values were consistent with the relative abundance of the correlated OTUs of particle size, which were 0.9, 34.2, and 0.9% at sites 2, 4, and 5, respectively. As the distribution of particle size approached the sediment depth direction, the relative abundance of the correlated OTUs of particle size increased. The above results indicated that the correlations among particle size and its correlated OTUs were probably pseudo correlations. At least, particle size was not the main effective environmental factor for sediment microbial community structure in Chaohu Lake.

For particle evenness, from sampling sites 2 to 5, the relative abundance of its correlated OTUs was 1.5, 15.3 and 1.5%, respectively. They were also consistent with the Spearman correlation coefficients between particle evenness and sediment depth, which were 0.61, −0.75, and 0.42 at each sampling site. Since the abundance of the correlated OTUs was only abundant at sampling site 4, the question was also raised about whether the correlations between particle evenness and its correlated OTUs were true. According to Figure 4, except for sampling site 4, the components of the correlated phyla of particle evenness were different from each other, both in the kinds and contributions of phyla. Given that the relative abundances of the correlated OTUs of particle evenness were at an exceptionally low level at sites 2, and 5, the above analysis supports the premise that particle evenness was also not the main effective environmental factor for the sediment microbial communities in Chaohu Lake.

### Analysis of volumetric water content and total interstitial space with their correlated OTUs

For volumetric water content and total interstitial space, they all have a large amount of correlated OTUs (Figure 4). RDA analysis in Figure 7 also showed that they may have effects on microbial community structure (axis length). However, in Figure 4, the similarities of the correlated OTUs in abundance and phyla kinds required further discussion between the two factors and sediment depth. (Sediment depth was not the main environmental gradient at site 3, so comparison at site 3 has been removed.)

According to Figure 4, the correlated phyla of volumetric water content and their abundance were like the phyla correlated with sediment depth. In addition, in Figures 4, 6, their similarities (kinds and contributions of correlated phyla) obviously decreased from sampling site 5 to site 4, and site 2. This tendency was in accordance with the methane emission characteristics mentioned above. Thus, correlations between volumetric water content and its abundant correlated OTUs probably resulted from the similar distributions between volumetric water content and sediment depth. Meanwhile, this similar distribution may be significantly affected by excess methane emissions.

To confirm the above assumptions, there were three points. First, the correlative OTUs of volumetric water content was more abundant at the sampling sites where volumetric water content was correlated with sediment depth. From sampling sites 2 to 5, Spearman correlation coefficients between volumetric water content and sediment depth were − 0.93, −0.91, and − 0.90, respectively. Meanwhile, the relative abundances of the correlated OTUs of volumetric water content were 37.8, 39.8, and 52.2% at each sampling site. For sediment depth, the relative abundances of its correlated OTUs were 41.4, 54.8, and 62.2% at sites 2, 4 and 5, respectively. Second, as it showed, the correlative OTUs of volumetric water content were far less than the correlative OTUs of sediment depth at all sampling sites. Third, at sampling site 3, Volumetric water content nearly has no corelative OTUs, when it was uncorrelated with sediment depth. This implied that the effects of volumetric water content on microbial community structure were far less than the main environmental gradient.

Similar to volumetric water content, the total interstitial space has similar kinds of correlated phyla with sediment depth (Figure 4, main phyla: Proteobacteria, Acidobacteria, Chloroflexi, Bacteroidetes, Planctomycetes, etc.). From sampling sites 2 to 5 (site 3 was removed), the results of Spearman correlation analysis between total interstitial space and sediment depth were − 0.90, −0.90, and − 0.89, respectively. The correlated OTUs of the total interstitial space were also only abundant at the sites where the total interstitial space was correlated with sediment depth. Besides, the correlated OTUs of the total interstitial space were also far less than the correlated OTUs of sediment depth at all sites. Total interstitial space has nearly none correlated OTUs at the site 3. Thus, it was probably not the main factor for microbial community structure.

Overall, for volumetric water content and total interstitial space, at the sampling sites (2, 4, and 5), their correlations with some OTUs were probably the pseudo correlations, because of the similar distributions with the main environmental gradients.

## Conclusion

Overall, among these six space parameters, sediment depth was the closest one to the main environmental gradient for sediment microbial community structure. Then, this study discussed whether five sediment space were effective in affecting microbial community structure in Chaohu Lake sediments. The conclusions were that, gas space which caused by excess methane emissions can promote the mixture of microbial communities in different layers. However, including gas space, all these five environmental factors, they were ineffective for affecting the microbial community structure in a sediment layer.

## Data availability statement

The datasets presented in this study can be found in online repositories. The names of the repository/repositories and accession number(s) can be found in the article/Supplementary material.

## Author contributions

XL and AR: conceptualization and methodology. XL, XZ, YX, ZW, SL, and AR: investigation. XL: software, data curation, visualization, and writing – original draft. XL, CS, and AR: writing, review, and editing. AR and XZ: funding acquisition. All authors contributed to the article and approved the submitted version.

## Funding

This work was supported financially by the National Natural Science Foundation of China (No. 42077221), Independent Research Project of State Key Laboratory of Hydrology and Water Resources (No. 20165042412) and the Postgraduate Research & Practice Innovation Program of Jiangsu Province (KYCX20_0458).

## Conflict of interest

The authors declare that the research was conducted in the absence of any commercial or financial relationships that could be construed as a potential conflict of interest.

## Publisher’s note

All claims expressed in this article are solely those of the authors and do not necessarily represent those of their affiliated organizations, or those of the publisher, the editors and the reviewers. Any product that may be evaluated in this article, or claim that may be made by its manufacturer, is not guaranteed or endorsed by the publisher.
