# Supplementary material for: Whether interstitial space features were the main factors affecting sediment microbial community structures in Chaohu Lake
Source: Front Microbiol. 2022 Dec 14;13:1024630. doi: 10.3389/fmicb.2022.1024630 (PMC9796575; doi:10.3389/fmicb.2022.1024630)
Supplement: Supplementary file 1 [file Table_1.DOCX]

***Frontiers in Microbiology***

*Supporting information for*

**Whether the interstitial space features were the main factors for affecting sediment microbial community structures in Chaohu Lake**

**Xiang Lu^1,2^, Xiaotian Zhou^1,2^, Christian von Sperber^3^, Yaofei Xu^1,2^, Zhipeng Wei^1,2^, Aidong Ruan^1,2*^**

*^1^ State Key Laboratory of Hydrology-Water Resources and Hydraulic Engineering, Hohai University, Nanjing 210098, China.*

*^2^ College of Hydrology and Water Resources,**Hohai University, Nanjing 210098, China.*

*^3^McGill University, Montreal QC H3A 0B9, Canada*

**^*^Corresponding Authors:**

Name: Aidong Ruan E-mail: [adruan@hhu.edu.cn](mailto:adruan@hhu.edu.cn)

**Contents of this file:**

Table S1

**Additional Supporting Information:**

Table S1 The detailed information about sequencing data quality control and the taxonomy of OTUs annotation results

**Brief Introduction:**

The supporting information of this file are mainly about the process of controlling the quality of sequence data and summary of OTUs annotation.

Table S1 The detailed information about sequencing data quality control and the taxonomy of OTUs annotation results

| Sampling site | Sediment depth | PE reads | Raw Tags | Clean Tags | Effective Tags | OTUs Tags  >0.005% | OTUs | Taxonomy | | | | | | | | | | | | |
| --- | --- | --- | --- | --- | --- | --- | --- | --- | --- | --- | --- | --- | --- | --- | --- | --- | --- | --- | --- | --- |
|  |  |  |  |  |  |  |  | Kingdom | | Phylum | | Class | | Order | | Family | | Genus | | Species |
| 2 | 2.5 | 79897 | 77079 | 76007 | 72435 | 37679 | 1644 | 2 | | 42 | | 98 | | 206 | | 310 | | 451 | | 456 |
|  | 7.5 | 79742 | 76685 | 75496 | 73412 | 44745 | 1742 | 2 | | 44 | | 101 | | 212 | | 319 | | 465 | | 471 |
|  | 12.5 | 79877 | 76673 | 75501 | 74206 | 48478 | 1793 | 2 | | 43 | | 101 | | 213 | | 321 | | 463 | | 472 |
|  | 17.5 | 79920 | 76869 | 75746 | 73779 | 41800 | 1894 | 2 | | 49 | | 109 | | 222 | | 327 | | 470 | | 478 |
|  | 22.5 | 80060 | 76675 | 75465 | 74506 | 51803 | 1982 | 2 | | 49 | | 110 | | 226 | | 342 | | 490 | | 499 |
|  | 27.5 | 80131 | 77368 | 76198 | 75441 | 54495 | 2048 | 2 | | 51 | | 113 | | 234 | | 355 | | 512 | | 522 |
|  | 32.5 | 79905 | 77296 | 76086 | 75571 | 54035 | 2050 | 2 | | 51 | | 115 | | 239 | | 354 | | 514 | | 524 |
|  | 37.5 | 80057 | 72039 | 70669 | 69809 | 46226 | 1801 | 2 | | 51 | | 110 | | 218 | | 322 | | 452 | | 461 |
|  | 42.5 | 79848 | 77134 | 75940 | 75687 | 57037 | 1959 | 2 | | 51 | | 112 | | 230 | | 342 | | 479 | | 489 |
| 3 | 2.5 | 80176 | 77364 | 76214 | 72747 | 35973 | 1659 | 2 | | 43 | | 96 | | 201 | | 297 | | 436 | | 440 |
|  | 7.5 | 80177 | 77029 | 75920 | 72449 | 41501 | 1765 | 2 | | 46 | | 103 | | 207 | | 313 | | 461 | | 468 |
|  | 12.5 | 79717 | 77092 | 75987 | 73503 | 43065 | 1770 | 2 | | 44 | | 100 | | 210 | | 319 | | 459 | | 465 |
|  | 17.5 | 79754 | 77081 | 75844 | 73282 | 44740 | 1810 | 2 | | 46 | | 104 | | 214 | | 322 | | 467 | | 475 |
|  | 22.5 | 80153 | 77149 | 75819 | 73711 | 46045 | 1867 | 2 | | 49 | | 108 | | 217 | | 325 | | 467 | | 476 |
|  | 27.5 | 79431 | 76624 | 75278 | 73444 | 49420 | 1729 | 2 | | 41 | | 97 | | 202 | | 314 | | 456 | | 465 |
|  | 32.5 | 80251 | 77251 | 75829 | 74310 | 51762 | 1720 | 2 | | 45 | | 102 | | 211 | | 315 | | 455 | | 463 |
|  | 37.5 | 80599 | 77589 | 76251 | 74294 | 44425 | 1692 | 2 | | 42 | | 100 | | 204 | | 310 | | 440 | | 448 |
|  | 42.5 | 80071 | 77159 | 75792 | 74201 | 45854 | 1843 | 2 | | 47 | | 107 | | 217 | | 321 | | 456 | | 462 |
| Sampling site | Sediment depth | PE reads | Raw Tags | Clean Tags | Effective Tags | OTUs Tags  >0.005% | OTUs | Taxonomy | | | | | | | | | | | | |
|  |  |  |  |  |  |  |  | Kingdom | Phylum | | Class | | Order | | Family | | Genus | | Species | |
| 4 | 2.5 | 80150 | 77354 | 75945 | 73035 | 35496 | 1779 | 2 | 44 | | 103 | | 210 | | 308 | | 451 | | 456 | |
|  | 7.5 | 79994 | 77268 | 76076 | 74566 | 46055 | 1972 | 2 | 49 | | 110 | | 227 | | 336 | | 479 | | 488 | |
|  | 12.5 | 80380 | 77479 | 76245 | 74953 | 50113 | 1798 | 2 | 48 | | 109 | | 217 | | 322 | | 448 | | 457 | |
|  | 17.5 | 79917 | 77249 | 75967 | 75063 | 53792 | 1838 | 2 | 47 | | 106 | | 214 | | 314 | | 441 | | 449 | |
|  | 22.5 | 80092 | 76997 | 75678 | 75341 | 57836 | 1792 | 2 | 51 | | 110 | | 217 | | 316 | | 435 | | 442 | |
|  | 27.5 | 80322 | 77349 | 76026 | 75844 | 59303 | 1546 | 2 | 48 | | 107 | | 210 | | 294 | | 395 | | 402 | |
|  | 32.5 | 79878 | 76929 | 75605 | 75282 | 56152 | 1455 | 2 | 49 | | 105 | | 202 | | 280 | | 371 | | 378 | |
|  | 37.5 | 80344 | 77169 | 75789 | 75552 | 58801 | 1444 | 2 | 48 | | 104 | | 204 | | 285 | | 373 | | 380 | |
|  | 42.5 | 79712 | 77029 | 75720 | 75463 | 59163 | 1382 | 2 | 49 | | 104 | | 197 | | 276 | | 358 | | 364 | |
| 5 | 2.5 | 79818 | 77300 | 76224 | 73983 | 38824 | 1596 | 2 | 46 | | 101 | | 203 | | 300 | | 434 | | 439 | |
|  | 7.5 | 80048 | 77219 | 76090 | 75337 | 54325 | 1689 | 2 | 48 | | 103 | | 211 | | 319 | | 453 | | 458 | |
|  | 12.5 | 79793 | 77357 | 76317 | 74942 | 47590 | 1669 | 2 | 49 | | 106 | | 213 | | 307 | | 430 | | 436 | |
|  | 17.5 | 80097 | 77487 | 76373 | 75770 | 58029 | 1667 | 2 | 45 | | 104 | | 208 | | 305 | | 427 | | 432 | |
|  | 22.5 | 79955 | 77227 | 76131 | 75260 | 52517 | 1775 | 2 | 49 | | 108 | | 218 | | 315 | | 439 | | 444 | |
|  | 27.5 | 80040 | 77299 | 76113 | 75746 | 57594 | 1777 | 2 | 49 | | 112 | | 224 | | 326 | | 446 | | 453 | |
|  | 32.5 | 79995 | 76904 | 75616 | 75524 | 62704 | 1503 | 2 | 47 | | 106 | | 212 | | 305 | | 414 | | 420 | |
|  | 37.5 | 80092 | 77523 | 76307 | 76197 | 62925 | 1282 | 2 | 45 | | 99 | | 197 | | 282 | | 378 | | 385 | |
|  | 42.5 | 79920 | 76973 | 75730 | 75650 | 63580 | 1182 | 2 | 45 | | 98 | | 194 | | 271 | | 366 | | 371 | |

Note: PE Reads, the number of initial sequencing reads. Raw tags: the number of raw sequence data after merged. Clean Tags: the number of sequence data after the filter of Trimmomatic v0.33. Effective Tags: the number of sequence data after the filter of UCHIME v4.2. OUT tags: the number of sequence data after removing the OTUs whose relative abundance less than 0.005%.
